# Atherogenic index of plasma is associated with vertebral fracture: a longitudinal study

**DOI:** 10.3389/fendo.2025.1540558

**Published:** 2025-05-28

**Authors:** Xin Zhang, Pinliang Xie, Yong Yin, Xinfeng Li

**Affiliations:** Department of Orthopedics, Jiading District Central Hospital Affiliated Shanghai University of Medicine & Health Sciences, Shanghai, China

**Keywords:** atherogenic index of plasma, fracture, spine, longitudinal study, general population

## Abstract

**Purpose:**

The atherogenic index of plasma (AIP), recognized as a marker of atherosclerosis, which also has a profound impact on bone metabolism. However, research exploring the association between the AIP and the probability of vertebral fractures in populations is still relatively scarce. The study aims to evaluate the association between the AIP and vertebral fractures probability in individuals in a longitudinal study.

**Patients and methods:**

A total of 1395 subjects who were older than 55 years and underwent CT scans for lung cancer screening between July 2019 and July 2021 were enrolled and followed up for a duration ranging from 8 months to 6 years. Among them, 91 individuals experienced new vertebral fractures. Participants were stratified into four groups based on AIP quartiles. The association between the AIP and vertebral fractures probability was then assessed by cox proportional hazards model.

**Results:**

The incidence of vertebral fracture decreased with increasing AIP (p for trend = 0.001). Kaplan-Meier survival analysis indicated that vertebral fractures were more likely to occur in patients with low levels of AIP (log-rank, all P < 0.05). Multivariate Cox regression analysis showed that AIP was negatively associated with the probability of vertebral fractures even after accounting for confounding factors (adjusted hazard ratio (aHR) = 0.27, 95%CI = 0.10-0.71 for continuous AIP; aHR = 0.48, 95%CI = 0.26-0.90 for Q2; aHR = 0.41, 95%CI = 0.19-0.88 for Q4, respectively). Subgroup analysis showed that such associations were mainly observed in male subjects. Restricted cubic splines further showed that the probability of vertebral fracture decreased with the increasing of AIP after adjusting with confounders in overall population and men, but not in women.

**Conclusion:**

Our study demonstrated a strong association between the AIP and the probability of vertebral fracture. Low AIP may be an associated factor of vertebral fracture.

## Introduction

Blood lipids, including triglycerides (TG), total cholesterol (TC), high-density lipoprotein cholesterol (HDL-c), and low-density lipoprotein cholesterol (LDL-c), have been implicated in bone metabolism. Studies suggest that dysregulation in lipid metabolism can lead to bone metabolic disorders, potentially causing conditions such as osteoporosis, bone loss, and bone fractures (1–4). However, the association between lipids levels and bone metabolism is complicated. Some studies indicate that higher levels of HDL-c are associated with a lower risk of osteoporotic fractures (5). However, opposite result was also reported. Hussain et al. showed that higher levels of HDL-c are associated with an increased fracture risk (6). Conversely, elevated levels of LDL-c and TG have been linked to an increased risk of fractures, indicating a potential negative impact on bone health (7, 8).

The atherogenic index of plasma (AIP) was defined as the base 10 logarithm of the ratio of the molar concentration of triglycerides (TG) to HDL-c. Considering both TG and HDL-c levels may be more accurately reflecting dyslipidemia’s (9). AIP is a valuable tool in cardiovascular disease risk and prognosis assessment (10, 11). It provides a measure of the balance between the potentially atherogenic lipoproteins and the cardioprotective HDL-c. A higher AIP value indicates a greater risk of developing atherosclerosis and related cardiovascular events. Monitoring AIP can help in the management of cardiovascular health. Some studies also showed that AIP is related to metabolism diseases, such as diabetes (12, 13) and metabolic syndrome (14, 15).

Interestingly, several studies have shown that AIP was related to bone metabolism. Xu reported that femur BMD and lumbar spine BMD were both increased with increasing AIP (16). However, He and colleagues reported completely opposite results. They observed a negative correlation between the AIP and total BMD (17). A study also showed that AIP is related to a degraded trabecular bone score (TBS) which is an important biomarker of bone fracture (18). However, to our knowledge, the association between AIP and fracture in general population has not been clarified. In the present longitudinal study, we investigated such association in a population who underwent CT lung cancer screening.

## Methods

### Subjects

CT lung cancer screening was recommended to subjects aged 50–80 years (19). We confirmed 1874 individuals who were older than 55 years and underwent clinical CT scans of the chest or lumbar spine for lung cancer screening between 2019 July and July 2021. A total of 1395 participants were identified and followed up for 8 months to 6 years. The exclusion criteria are as follows: (1) received only one time scan or lost to follow-up; (2) missing demographic, clinical and laboratory information; (3) poor imaging quality for fracture evaluation; (4) spine fracture at baseline; (5) history of metabolic bone diseases, cancer, rheumatic diseases, and severe kidney and liver dysfunction. Two radiologists determine whether a new fracture has occurred by observing the baseline and follow-up radiographic images. Consequently, 91 patients in the study developed new osteoporotic fractures during the follow-up period. The Ethics Committee of the Jiading District Central Hospital approved this trial’s conduct. Informed consent was waived because of the retrospective design.

### Data collection

The data, which included demographic information, such as age and sex, body mass index (BMI), and laboratory biochemical indicators were collected. The following biochemical indicators were obtained from the medical system records: aspartate aminotransferase (AST), serum creatinine, fasting blood glucose level, serum TG, TC, HDL-c, LDL-c, serum albumin, fasting blood glucose level. AIP was calculated using the following equation: log10 [TG(mg/dL)/HDL-c (mg/dL)].

### Bone CT assessment

Trabecular bone CT attenuation was used as indicator of bone mass. All CT scans were performed in one CT (GE Optima CT680, USA). The scan protocol was as follows: voltage of 120 kV, 100–120 mAs, thickness of 0.625 mm. The CT images were reconstructed in the workstation using 0.625-mm section thickness and 0.5-mm increments. A 2–3 cm^2^ region of interest (ROI) was placed in the cancellous bone region of the medium level of T12 and L1 vertebral body. For each measurement, we avoided cortical bone, the posterior venous plexus, bone islands, and other heterogeneous areas. The mean CT attenuation of T12 and L1 was calculated and used for analyses. Incident vertebral fractures was defined as any vertebra that was graded as normal (grade 0) at baseline and exhibited at least mild deformation (grades 1-3, or a reduction in height of approximately 20-25%) at follow-up based on Gennant’s classification. All individuals underwent CT scans for lung cancer screening at baseline.

### Statistical analysis

All statistical analyses were conducted using SPSS version 26.0 or R version 4.1.2. Continuous variables with normal distributions are expressed as mean (± SD), while those with non-normal distributions are presented as median (interquartile ranges). Categorical variables are represented by frequencies. The chi-square test or Fisher’s exact test was employed to assess differences between groups for categorical variables, whereas independent samples t-tests or the Mann-Whitney U test were utilized for continuous variables. According to the quartiles of the AIP, participants were divided into four groups: Q1 (< 0.10), Q2 (0.1-0.30), Q3 (0.30-0.50) and Q4 (> 0.50). Cox proportional hazards model was applied to evaluate the association between the AIP and the risk of vertebral fractures, yielding adjusted hazard ratios (aHRs) with 95% confidence intervals (CIs) for each indicator. Then, three multivariate Cox models were built and used to gradually adjust for potential confounding factors for a fragility fracture endpoint event. Additionally, cumulative hazard curves are presented. Participants were stratified by sex to examine sex-specific associations. The restricted cubic splines analysis was used to illustrate the multivariable-adjusted hazard ratios for the risk of vertebral fracture across varying levels of the AIP. A P value of less than 0.05 was considered statistically significant.

## Results

### Baseline characteristics

The baseline characteristics are given in **Table 1**. The study group included 1395 individuals. Significant differences were observed in age, sex, bone CT attenuation (HU), AST, albumin, blood glucose and serum lipids levels (TC, TG, HDL-c and LDL-c) among different AIP groups (all p < 0.01). The incidence rates of vertebral fracture were related to the levels of the AIP (fracture: 10.5%, 4.8%, 8.3% and 3.8% for Q1, Q2, Q3 and Q4 of the AIP, respectively; p for trend = 0.001).

**Table 1:** Characteristics of the subjects.

Variables	AIP quartiles				
	Q1 (n = 315)	Q2 (n = 368)	Q3 (n = 345)	Q4 (n = 367)	P
Age (years)	67.65 ± 8.32	66.81 ± 8.21	67.23± 8.08	65.53 ± 7.48	0.004
Sex (women/men)	157/158	148/220	137/208	131/236	0.002
BMI (kg/m^2^)	24.15 ± 3.03	25.33 ± 2.48	26.28 ± 2.62	27.12 ± 2.13	< 0.001
Bone CT attenuation (HU)	125.8 ± 39.25	126.2 ± 36.25	131.7 ± 40.54	134.7± 38.23	0.005
AST (U/L)	24.24 ± 8.40	23.26 ± 6.67	24.00 ± 7.35	26.11 ± 9.95	0.011
Albumin (g/L)	40.03 ± 3.23	40.48 ± 2.98	40.55 ± 3.01	40.90 ± 3.20	0.004
Creatinine (μmol/L)	76.78 ± 35.07	78.08 ± 15.81	79.54 ± 17.78	81.24 ± 31.99	0.14
HDL-c(mmol/L)	1.82± 0.34	1.56 ± 0.30	1.41 ± 0.26	1.25 ± 0.23	< 0.001
LDL-c(mmol/L)	2.55 ± 0.70	2.91 ± 0.79	3.04 ± 0.91	3.03 ± 0.83	< 0.001
TC (mmol/L)	4.54 ± 0.94	4.74 ± 1.03	4.73 ± 1.13	4.86 ± 1.05	0.001
TG (mmol/L)	0.71 ± 0.19	1.10 ± 0.22	1.50 ± 0.31	2.62 ± 1.29	<0.001
AIP	-0.06 ± 0.13	0.20 ± 0.06	0.38 ± 0.05	0.66 ± 0.16	< 0.001
Diabetes	18 (5.7%)	33 (9.0%)	40 (12.2%)	45 (12.3%)	0.018
Fracture	33 (10.5%)	16 (4.3%)	28 (8.1%)	14 (3.8%)	0.001

AIP, atherogenic index of plasma; AST, aspartate aminotransferase; BMI, body mass index; CT, computed tomography; HDL-c, high-density lipoprotein cholesterol; HU, Hounsfield unit; LDL-c, low-density lipoprotein cholesterol; TC, total cholesterol; TG, triglyceride.

AIP quartiles, Q1, < 0.1; Q2, 0.1-0.3; Q3, 0.3-0.48; Q4, > 0.48.

### Cox proportional hazard models for the risk of vertebral fracture

**Figure 1:**
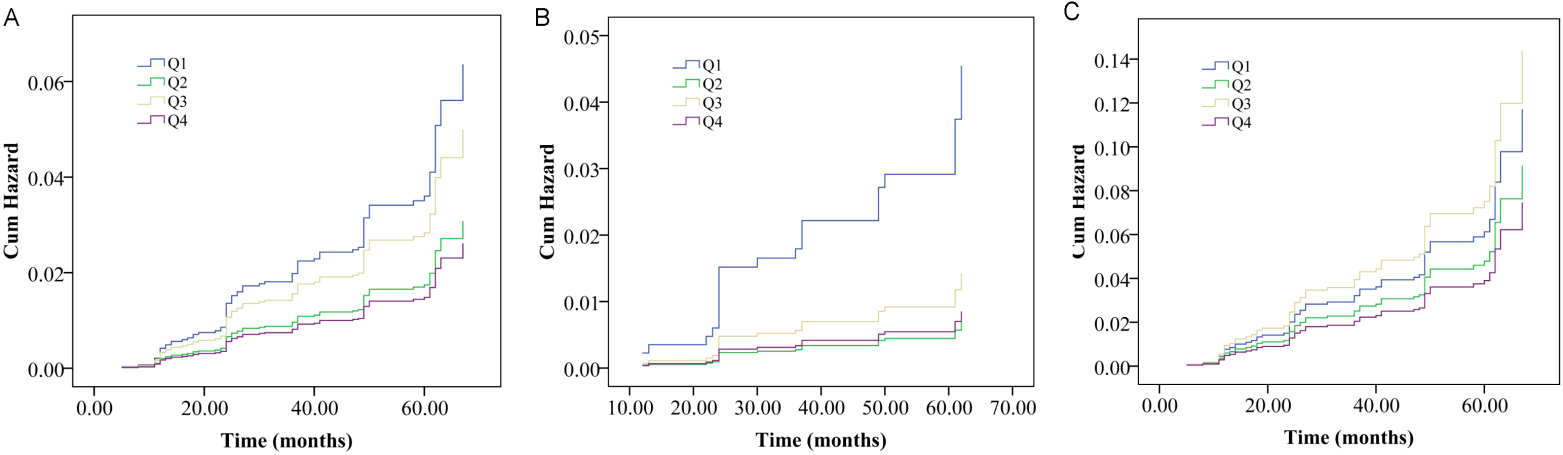
Cumulative probability curves of vertebral fracture probability divided by interquartile range of the atherogenic index of plasma (AIP) in overall population **(A)**, male **(B)** and female **(C)** populations.

The association of the AIP and vertebral fracture risk were firstly showed by cumulative hazard curve (**Figure 1**). During the follow-up period, out of 1395 subjects, 91 experienced vertebral fracture events (6.57%). In total population and female, the Kaplan-Meier survival curve analysis showed that compared with the participants with low level of AIP (Q1), those with Q2 and Q4 of AIP had a significantly decreased probability of vertebral fracture during follow-up (log-rank, all P < 0.05).

The association of the AIP and vertebral fracture probability was further evaluated by multivariate Cox regression analysis (**Table 2**). In model I which was adjusted with age, gender, bone CT attenuation and BMI, the higher AIP was associated with the lower probability of vertebral fracture (aHR = 0.49, 95%CI = 0.27-0.91 for Q2 and aHR = 0.48, 95%CI:0.23-0.99 for Q4) compared with low AIP (Q1). In fully adjusted model which was adjusted with age, gender, bone CT attenuation, BMI, diabetes, creatinine, albumin, AST and LDL-c, the negative association between the AIP and the probability of the vertebral fracture remained statistically significant respectively (aHR = 0.48, 95%CI = 0.26-0.90 for Q2; aHR = 0.41, 95%CI = 0.19-0.88 for Q4).

**Table 2:** Association between the atherogenic index of plasma and the risk of vertebral fracture.

Variables	Model 1	P	Model 2	P	Model 3	p
	aHR (95%CI)		aHR (95%CI)		aHR (95%CI)	
Age (years)	1.05 (1.02-1.09)	0.001	1.05 (1.02-1.09)	0.001	1.05 (1.02-1.09)	0.001
Gender (women vs men)	2.41 (1.45-4.00)	0.001	2.41 (1.45-4.00)	< 0.001	2.70 (1.59-4.60)	< 0.001
Bone CT attenuation (HU)	0.97 (0.96-0.98)	< 0.001	0.97 (0.96-0.98)	< 0.001	0.97 (0.96-0.98)	< 0.001
AIP	0.31 (0.12-83)	0.02	0.33 (0.12-0.87)	0.024	0.27 (0.10-0.71)	0.008
Q1 (< 0.1)	1		1		1	
Q2 (0.1-0.3)	0.49 (0.27-0.91)	0.023	0.50 (0.27-0.92)	0.026	0.48 (0.26-0.90)	0.021
Q3 (0.3-0.5)	0.83 (0.47-1.46)	0.98	0.83 (0.47-147)	0.52	0.79 (0.45-1.39)	0.41
Q4 (> 0.5)	0.48 (0.23-0.99)	0.048	0.49 (0.23-1.00)	0.05	0.41 (0.19-0.88)	0.02

Model 1 was additionally adjusted with body mass index; Model 2 was further adjusted for diabetes. Model 3 was additionally adjusted for serum albumin, creatinine, aspartate aminotransferase and low-density lipoprotein cholesterol.

aHR, adjusted hazard ratio; CI, confidence interval; AIP, atherogenic index of plasma.

**Figure 2:**
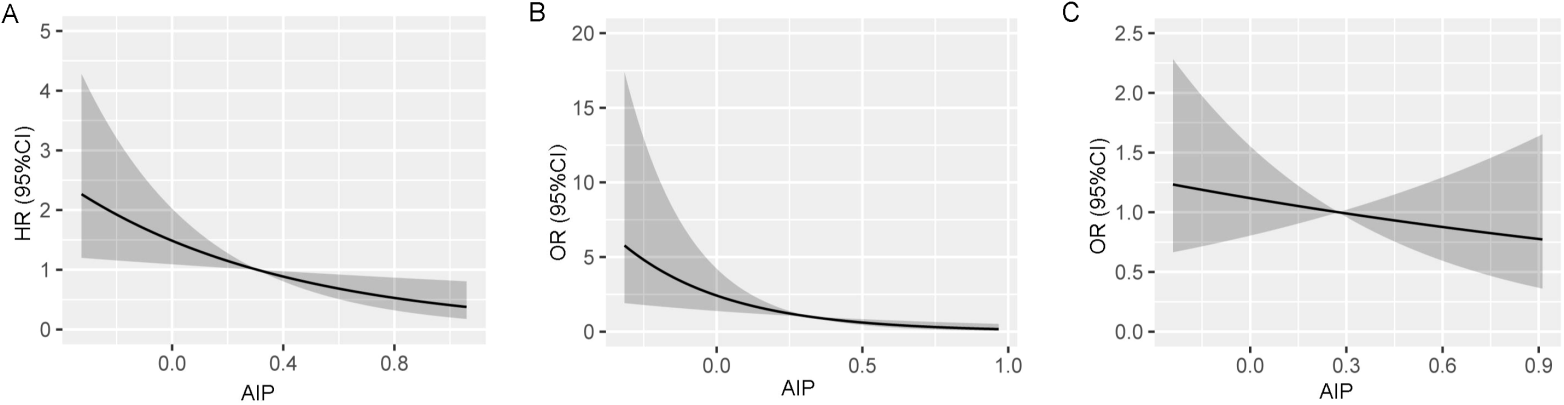
Restricted cubic splines show the multivariable adjusted hazard ratio for the probability of vertebral fracture according to the level of atherogenic index of plasma (AIP) in the overall population **(A)**, male **(B)** and female **(C)** populations. Age, sex (overall population), body mass index, serum albumin, creatinine, aspartate aminotransferase, low-density lipoprotein cholesterol, and bone attenuation or diabetes were adjusted.

Restricted cubic splines further showed that the probability of vertebral fracture decreased with the increasing of AIP after adjusting with confounders (**Figure 2A**).

### Subgroup analysis

Subsequently, we showed the association between AIP and the probability of vertebral fracture in men and women, respectively (**Table 3**). No significant associations were found between AIP and probability of vertebral fracture in all the three models in women (aHR = 0.79, 95%CI: 0.38-1.63 for Q2, aHR = 1.23, 95%CI: 0.62-2.43 for Q3 and aHR = 0.65, 95%CI: 0.26-1.66 for Q4). For male population, the association between AIP and probability of vertebral fracture was similar with the overall population. In model I which was adjusted with age, gender, bone CT attenuation and BMI, the higher AIP was associated with the lower probability of vertebral fracture (adjusted hazard ratio (aHR) = 0.16,95%CI = 0.04-0.56 for Q2, aHR = 0.33, 95%CI:0.11-0.99 for Q3 and aHR = 0.19, 95%CI:0.05-0.71) compared with low AIP (Q1). In fully adjusted Model, the probability of the vertebral fracture remained statistically significant (aHR = 0.15, 95%CI=0.04-0.56 for Q2; aHR = 0.19, 95%CI = 0.05-0.70 for Q4).

**Table 3:** Association between the atherogenic index of plasma and the risk of vertebral fracture divided by sex.

Sex	Quartiles	Model 1	P	Model 2	P	Model 3	p
		aHR (95%CI)		aHR (95%CI)		aHR (95%CI)	
Women	AIP	0.76 (0.24-2.35)	0.63	0.79 (0.25-2.48)	0.69	0.60 (0.18-2.00)	0.40
	Q1 (< 0.1)	1		1		1	
	Q2 (0.1-0.3)	0.81 (0.40-1.66)	0.57	0.82 (0.40-1.67)	0.60	0.79 (0.38-1.63)	0.52
	Q3 (0.3-0.5)	1.33 (0.69-2.58)	0.40	1.34 (0.69-2.59)	0.39	1.23 (0.62-2.43)	0.55
	Q4 (> 0.5)	0.80 (0.34-1.91)	0.62	0.83 (0.34-1.99)	0.67	0.65 (0.26-1.66)	0.37
Men	AIP	0.07 (0.01-0.38)	0.002	0.07 (0.01-0.39)	0.002	0.07(0.01-0.40)	0.003
	Q1 (< 0.1)	1		1		1	
	Q2 (0.1-0.3)	0.16 (0.04-0.56)	0.004	0.16 (0.04-0.57)	0.005	0.15 (0.04-0.56)	0.004
	Q3 (0.3-0.5)	0.33 (0.11-0.99)	0.047	0.34 (0.12-1.01)	0.052	0.32 (0.10-1.02)	0.051
	Q4 (> 0.5)	0.19 (0.05-0.71)	0.013	0.20 (0.05-0.71)	0.013	0.19 (0.05-0.70)	0.013

Model 1 was adjusted with age, bone mass, and body mass index; Model 2 was further adjusted for diabetes. Model 3 was additionally adjusted for serum albumin, creatinine, aspartate aminotransferase and low-density lipoprotein cholesterol.

aHR, adjusted hazard ratio; CI, confidence interval; AIP, atherogenic index of plasma.

Restricted cubic splines further showed that the probability of vertebral fracture decreased with the increasing of AIP after adjusting with confounders in men (**Figure 2B**), but not in women (**Figure 2C**).

## Discussion

Associations of serum lipid levels and fracture risk have been reported. However, the results are conflicting. Moreover, the association between AIP and fracture probability has not been well studied. This longitudinal study in a Chinese population is the first report which showed that AIP was associated with the probability of vertebral fracture. However, such associations were mainly observed in overall population and male subjects, but not female subjects. The probability of vertebral fracture was 52% lower in subjects with high AIP (≥ 0.50) than in those with low AIP (< 0.10). This study reported a novel associated factor for vertebral fracture, which may be useful for detecting high fracture probability early and fracture management.

Fractures are the most serious clinical outcome of osteoporosis. Interestingly, some studies also suggest a link between blood lipid levels and the risk of fractures. A meta-analysis indicated that serum TC concentration is positively correlated with the risk of fractures (20). A U-shaped relationship between high-density lipoprotein cholesterol (HDL-c) and hip fractures has been reported (21). In contrast, a recent study showed an association between HDL-c levels and the risk of fractures in the age group of 60 years and above, but not in the age group below 60 (22). Similar results have been reported in healthy elderly individuals over the age of 65 (6). The association between TG and fracture risk was also reported in women (23). However, those studies mainly focused on single lipids marker. Considering the different role of TG and HDL-c, it would be better to combine them together to obtain a new comprehensive index and show its role in fracture. Interestingly, AIP had been reported in literature and many studies have shown the association of AIP and cardiovascular diseases (10, 11, 24). The association between AIP and metabolic diseases was also reported (12, 13). Two recent studies also found that AIP was associated with bone mineral density (16, 17). However, no study has shown the link between AIP and fracture probability. Our study showed that high AIP was a protective factor for spine fracture, especially for men. Our data demonstrated that AIP should be considered during the fracture management in older adults.

For overall population, AIP at Q3 did not show statistical significance compared to that in Q1. But the OR was still lower than 1.0, which also indicated that a low risk of fracture in Q3 group. We speculated that the association may be sex-dependent. Therefore, we performed subgroup analysis. The data of subgroup analysis confirmed that the association was sex-dependent. Why Q3 did not show statistical significance compared to Q1 in overall population may be affected by sex. Although AIP at Q3 also did not show statistical significance in fully adjusted model compared to Q1 in male population, the OR was 0.32, which also indicated that a low risk of fracture in Q3 group. Moreover, the sample size of our study was relatively small which may affect the statistical power. The restricted cubic splines showed that fracture probability decreased by increasing of AIP. The relationship could be seen as a monotonic relationship between AIP and the number of fractures observed in male population. Why there was a sex-difference was unclear. One possible reason may be the high prevalence of osteoporosis in older women. Osteoporosis may play a more critical role for fracture in women. Interestingly, a recent study also showed that association of HDL-c and fracture risk were more common in men compared to women (25).

How AIP affects the risk of spine fracture is not understood. One of possible reason is that the positive relationship between HDL-c and fracture. A subjects with a high HDL-c may have a lower AIP because AIP was calculated by the 10 logarithm of the ratio of the TG to HDL-c. Another reason is that great level of TG may be associated with high bone mineral density (26). High bone mineral density will cause low probability of fracture. Moreover, AIP can be used as an indicator of atherosclerosis. A high AIP means a high risk of atherosclerosis. Blood vessels play a role in osteogenesis and osteoporosis (27). The atherosclerosis in bone arteries will result in bone loss. Consequently, the probability of fracture increased. High atherosclerosis burden is also associated with high inflammatory status (28, 29) which may be related excessive bone resorption. It has been shown that subclinical atherosclerotic cardiovascular disease is associated with high risk of hip fracture (30).

Our study has several advantages. This was a longitudinal study with relatively large sample size. In addition, to our knowledge, the present study may be the first to show the association of AIP and fracture probability in general population. Several limitations should be acknowledged. First, our study reported only the association between AIP and vertebral fracture probability. whether such associations exist between AIP and nonspine fractures has not been studied. Second, our study only simply discussed the probable mechanisms. The underlying cellular or molecular mechanisms were not studied. Third, although several variables were adjusted, other factors, such as drinking or smoking habits, physical exercise, were not controlled. Fourth, our study had the unequal sample size of male and female population. One possible reason for the small number of female subjects was the high prevalence of osteoporosis and vertebral fracture. More women was excluded at baseline because of the osteoporotic vertebral fracture. Our study only included subjects without vertebral fracture at baseline. Finally, our population is Chinese population. The generalizability of our results should be validated in other populations.

In summary, this study revealed that the AIP were independently associated with the probability of vertebral fracture. Our study reported a novel associated factor of bone fracture. Monitoring AIP may be useful for identifying subjects with high-risk fractures earlier. However, further studies are needed to reveal the mechanisms by which AIP affects bone metabolism and fracture.

## Data Availability

The original contributions presented in the study are included in the article/supplementary material. Further inquiries can be directed to the corresponding authors.
